# Pan-glioma analyses reveal species- and tumor-specific regulation of neuron-glioma synapse genes by lncRNAs

**DOI:** 10.3389/fgene.2023.1218408

**Published:** 2023-08-24

**Authors:** Wei Xiong, Xuecong Zhang, Bin Peng, Hao Zhu, Lijin Huang, Sha He

**Affiliations:** ^1^ Bioinformatics Section, School of Basic Medical Sciences, Southern Medical University, Guangzhou, China; ^2^ Neurosurgery Department, The Third Affiliated Hospital, Southern Medical University, Guangzhou, China

**Keywords:** gliomas, neuron-glioma synapse, pan-glioma analysis, lncRNA, species-specific

## Abstract

Gliomas are highly heterogeneous and aggressive. Malignant cells in gliomas can contact normal neurons through a synapse-like structure (called neuron-to-glioma synapse, NGS) to promote their proliferation, but it is unclear whether NGS gene expression and regulation show species- and tumor-specificity. This question is important in that many anti-cancer drugs are developed upon mouse models. To address this question, we conducted a pan-glioma analysis using nine scRNA-seq datasets from humans and mice. We also experimentally validated the key element of our methods and verified a key result using TCGA datasets of the same glioma types. Our analyses revealed that NGS gene expression and regulation by lncRNAs are highly species- and tumor-specific. Importantly, simian-specific lncRNAs are more involved in NGS gene regulation than lncRNAs conserved in mammals, and transgenic mouse gliomas have little in common with PDX mouse models and human gliomas in terms of NGS gene regulation. The analyses suggest that simian-specific lncRNAs are a new and rich class of potential targets for tumor-specific glioma treatment, and provide pertinent data for further experimentally and clinically exmining the targets.

## 1 Introduction

In humans and mice, 95% of protein-coding genes are conserved, but substantial lncRNA genes are species-specific; moreover, lncRNA expression is highly tissue-specific (Derrien et al., 2012; Necsulea et al., 2014; Lin et al., 2019). Further, many simian-specific lncRNA genes are expressed only in the brain (Derrien et al., 2012). These three factors make gene regulation by lncRNAs in the brain highly species- and tissue-specific. Since most drugs developed upon mouse models fail to cure human patients, revealing the species- and tissue-specificities and the underlying implications upon big data is important for glioma diagnosis and therapy. However, to what extent gene expression regulation by lncRNAs is species- and tissue-specific is poorly understood, especially in the brain and brain tumors.

Gliomas are highly aggressive and heterogeneous, and the two properties greatly influence glioma therapy and survival. High-grade gliomas are especially aggressive, with a 5-year survival rate of < 5% (Ostrom et al., 2014). Currently, all therapies (including surgical resections, radiotherapy, and chemotherapy) have very limited effects (Amarouch and Mazeron, 2005; Reitman et al., 2018), making identifying new therapeutic targets a pressing need. Increasing evidence indicates that interactions between cells in the tumor microenvironment (TME) play vital roles in glioma generation, migration, and responses to chemotherapy and immunotherapy (Osswald et al., 2015; Wang et al., 2017; Wu and Dai, 2017). A specific kind of intercellular interactions is between glioma cells and neuronal cells. NLGN3, a protein normally on the neuron surface, can be secreted as a mitogen and promote the growth and infiltration of glioma cells through the PI3K-mTOR pathway (Venkatesh et al., 2015). Multiple studies have confirmed that glioma cells can contact neurons through a synapse-like structure called neuron-to-glioma synapse (NGS), by which malignant cells mimic normal neurons and hijack potassium currents to promote their aggression and proliferation (Venkataramani et al., 2019; Venkataramani et al., 2022b; Venkatesh et al., 2019). Such synapses also form between the brain-metastatic breast cancer cells and neural cells to facilitate breast-to-brain metastasis (Zeng et al., 2019). Studies also demonstrate that NGS and neuronal activity-dependent paracrine contribute to forming neuron-tumor networks in gliomas (Venkataramani et al., 2022a). These discoveries indicate that the NGS is an important mechanism underlying glioma aggression and should be a key target for glioma therapy.

According to experimental studies, NGS formation and function rely on a set of genes involved in microtube formation, synaptogenesis, neurotransmitter receptors, and neuronal paracrine (hereafter termed NGS genes) (Venkatesh et al., 2019). The knockout of *NLGN3* (an NGS gene) can decrease the invasion of glioma *in vivo* in mouse models (Venkatesh et al., 2015), supporting that NGS genes can be therapeutic targets. However, researchers who use transgenic and patient-derived xenograft (PDX) mouse models to study tumors find that many drugs tested successfully in mice fail in human clinical trials (Mak et al., 2014; Gould et al., 2015). A previous study focusing on PDGF signaling in glioblastoma in humans, rats, mice, and dogs revealed that glioblastoma in these species shows highly distinct gene expression (Connolly et al., 2018). To explore NGS genes as targets for gliomas, more studies addressing the two questions are needed: whether NGS gene expression shows species- and tumor-specificity, and how the specificity is regulated, especially by lncRNAs (Yao et al., 2015; Alinejad-Rokny et al., 2020).

Pan-cancer analyses are highly valued in cancer studies. Many pan-cancer analyses have revealed shared features of different tumors (Ma et al., 2018; Campbell et al., 2020). Cross-species cancer analyses, which try to identify shared and species-specific features of tumors, have also drawn attention. Reported cross-species cancer analyses focused either on a specific aspect, such as PDGF signaling (Connolly et al., 2018), cancer genome (Robles-Espinoza and Adams, 2014), and cancer resistance-associated genes (Ulhas Nair et al., 2022), or on a specific cancer, such as pancreatic cancer (Elyada et al., 2019). To make the most of published data and to address the above-mentioned two questions, cross-species pan-gliomas analyses are required. In this study, we examined NGS genes and their potential regulation by lncRNAs using nine single-cell RNA-sequencing (scRNA-seq) datasets covering 5 glioma types in humans and mice. The examined gliomas include oligodendroglioma (Tirosh et al., 2016), anaplastic astrocytoma (Venteicher et al., 2017), medulloblastoma (Hovestadt et al., 2019; Cheng et al., 2020), glioblastoma (Neftel et al., 2019; Weng et al., 2019), and diffuse H3K27M glioma (Filbin et al., 2018), which range from the WTO II grade to the WTO IV grade. We found that NGS gene expression is highly tumor-specific and that multiple lncRNAs, especially simian-specific ones, critically regulate tumor-specific NGS genes. Importantly, we found that mouse and human gliomas have very limit similarities in NGS regulation by lncRNAs, including that transgenic mouse gliomas have little in common with PDX mouse models and human gliomas in terms of NGS gene regulation. We experimentally validated the key element of our methods and verified a key result using TCGA datasets of three glioma types. The combined TCGA and scRNA-seq data analysis reveals that malignant cells with NGS are indeed associated with worse survival. These results help identify the mechanisms behind gliomas’ species- and tumor-specificity and provide pertinent data for further experimentally and clinically identifying tumor-specific targets for glioma treatment.

## 2 Materials and methods

### 2.1 Data collection and pre-processing

Nine scRNA-seq datasets of gliomas (GSE131928, GSE89567, GSE70630, GSE102130, GSE119926, GSE122871, and GSE150752) were downloaded from the GEO website (Supplementary Table S1). GSE131928 contains two datasets that are adult and pediatric glioblastoma. GSE119926 contains two datasets that are human medulloblastoma and medulloblastoma PDX in mice. These datasets were generated by Drop-seq (GSE122871), 10X Genomics (GSE150752), and Smart-seq/Smart-seq2 (all others).

Batch effects may exist in samples of different studies and different sequencing protocols. Whether a researcher chooses to eliminate or not eliminate batch effects depends on the study’s primary purpose and the data’s property. Because gliomas are characterized by intra- and inter-tumoral heterogeneity, eliminating batch effects by integrating data using specific methods may risk removing real biological signals (including glioma heterogeneity). To preserve as much heterogeneity as possible between glioma samples, we did not remove the batch effect. However, we normalized each dataset using the *Seurat* (v3.6.1) package as follows (Satija et al., 2015).

For smart-seq datasets, transcripts per million (TPM) were first log-transformed using the equation $E_{ij}=\log_{2}\left(\frac{TPM_{ij}}{10}+1\right)$ where “i” refers to gene and “j” refers to cell. After the transformation, we used the *CreateSeuratObject* function in *Seurat* to read the E_ij_ expression matrix. For datasets generated by Drop-seq or 10X Genomics, we used the *read10X* function to read the UMI data directly. For all datasets generated by the three sequencing protocols, cells with *nFeature_RNA* < 3,000 and *nCount_RNA* < 4,000 were removed, all mitochondrial genes were removed, and each dataset was normalized using the *NormalizationData* function (with default parameters) to eliminate the effect of library size across cells. The numbers of remaining cells and genes in each dataset are listed in the (Supplementary Table S1). By default, the default parameters were used in the analyses described in the following sub-sections.

### 2.2 Cell clustering

For each dataset, the following steps were performed to cluster cells into clusters. First, we used the *FindVariableFeatures* function to identify the top 2000 highly variable genes, with the selection method set as “vst”. Second, all cells were scaled using the *ScaleData* function with the identified highly variable genes. Third, we used the *RunPCA* function to perform a linear dimension reduction. Fourth, we constructed the shared nearest neighbor (SNN) graph using the *FindNeighbors* function*,* with the number of principal components used for the *FindNeighbors* determined by the *ElbowPlot* function. Finally, we clustered cells using the *FindClusters* function (with data-specific resolution settings). This clustering method is based on optimizing SNN modularity. The data-specific resolution was 0.3 for pediatric glioblastoma in GSE131928, 0.8 for GSE122871, 0.8 for human medulloblastoma in GSE119926, 0.25 for GSE150752, 0.3 for GSE70630, and 0.4 for all other datasets. Cell clusters were visualized using t-distributed stochastic neighbor embedding (t-SNE) (using the *RunTSNE* function).

### 2.3 Identifying normal cell clusters

For each dataset, after cell clustering, the following steps were performed to identify normal cell clusters. First, we used *FindMarkers* function in *Seurat* to identify differentially expressed genes (DEGs) in each cluster compared to all cells in all other clusters (with “only.pos” = TRUE, “min.pct” = 0, “logfc.threshold” = 0.5, “test.use” = “wilcox”, and other parameters took the default values). Genes with logFC > 0.5 and adjusted *p*-value < 0.01 were defined as DEGs. Second, we applied gene set enrichment analysis to DEGs in each identified cell cluster to detect if any marker genes (i.e., DEGs) of normal cell types were enriched. Marker genes of normal cell types were collected from the *CellMarker* database (Zhang et al., 2019), and the gene set enrichment analysis was performed using *ClusterProfiler* (v3.14.3) (with default parameters) (Yu et al., 2012). If the DEGs of a cell cluster were significantly enriched in the marker genes of a cell type in the CellMarker database, this cell cluster was defined as this cell type (BH-adjusted *p*-value < 0.05).

### 2.4 Scoring cells using binary markers of normal cell clusters

After identifying normal cell clusters upon the enrichment of DEGs in annotated cell markers, we extracted the properties of each identified normal cell cluster to refine the identification of normal cells. First, we followed the approach developed by Hodge et al. (2019) to detect “binary markers” for each normal cell cluster. This method computed a binary score ranging from 0 to 1 to assess the exclusivity of a DEG between clusters. A higher binary score indicates greater exclusivity of gene expression in a particular cell cluster, and a DEG in a normal cell cluster with a binary score above 0.7 was defined as a binary marker of that normal cell cluster. Then, upon each set of binary markers, all cells in a dataset were scored using the *AddModuleScore* function. If a cell was scored above a dataset-specific cutoff, the cell was classified into the corresponding normal cell cluster.

### 2.5 Copy number analysis of cells

For each dataset generated by Smart-seq/Smart-seq2 and Drop-seq, we used a reported copy number analysis (CNA) method to evaluate whether a cell underwent genome duplication (Tirosh et al., 2016). First, we first sorted genes by chromosome and gene start position. Second, we used cells defined as normal cells upon the score of A*ddModuleScore* as the reference. Third, we computed the aggregate expression of each gene using the equation $E_{a}\left(i\right)=\log_{2}\left(average\left(\sum_{j=1}^{N}{TPM}_{ij}\right)+1\right)$ , where “i”, “j” and “N” refer to gene, cell, and the total number of cells, respectively. Only genes with $E_{a}$ > 4 (which indicates very high expression levels) were selected for further analysis. Fourth, for each selected gene, its relative expression in cell j was calculated by ${Er}_{ij}=E_{ij}-average\left(\sum_{j=1}^{N}E_{ij}\right)$ . Fifth, the initial CNA of each gene was averaged over the ${Er}_{ij}$ values of this gene, its 50 genes upstream, and its 50 genes downstream, and the CNA was denoted as CNAij=∑i−50i+50Erij/101 . Sixth, the baseline CNA of each gene was defined as the average of ${CNA}_{ij}$ of the reference cells and denoted as $Baseline\_{CNA}_{i}$ *.* Seventh, the relative CNA of each gene was calculated as ${CNA}_{ij}-Baseline\_{CNA}_{i}$ . Eighth, the CNA signal of each cell was defined as the averaged square of the relative CNA of all genes, that is, ${CNA\_Signal}_{j}=average\left(\sum_{i=1}^{M}{\left({CNA}_{ij}-Baseline\_{CNA}_{i}\right)}^{2}\right)$ , where “M” specifies the total number of genes. Finally, the CNA correlation of each cell was defined as the Pearson correlation between the relative CNA and the average of the relative CNA of non-reference cells from the same sample. The above CNA analysis was not applied to GSE119926 because the analysis needs the normal cells as the reference but no normal cell clusters were identified in GSE119926.

### 2.6 Identifying malignant cells

For datasets generated by Smart-seq/Smart-seq2 and Drop-seq, malignant cells were identified jointly upon three criteria: (a) A cell does not belong to normal cell clusters according to the cell cluster-based classification (see Method 2.3), (b) a cell is not defined as a normal cell when scored using the binary markers of normal cell clusters (see Method 2.4), (c) a cell has CNA signal > 0.02 and CNA correlation > 0.4 based on CNA analysis (see Method 2.5). For datasets generated by 10X genomics, malignant cells were identified based on the criteria (a) and (b) mentioned above. A cell was classified as malignant only when all the corresponding criteria gave concordant assignment. Since normal cell clusters were not identified in GSE119926, all cells in this dataset were defined as malignant cells.

### 2.7 Detection of NGS-negative and NGS-positive cells

We collected NGS genes from related studies (Osswald et al., 2015; Venkataramani et al., 2019; Venkatesh et al., 2019). Using the NGS genes as a signature, we first used *addmoduleScore* (with “nbin” = 30) in *Seurat* to score each cell to measure its potential to form NGS with neurons. Malignant cells with a score > 0 were identified as NGS-positive cells, which indicates they probably form NGS with neurons. Malignant cells with a score < 0 were identified as NGS-negative cells, which indicates they probably do not form NGS with neurons. By doing so, we divided malignant cells further into NGS-positive (NGS^+^) cells and NGS-negative (NGS^−^) cells.

### 2.8 Identifying malignant cells related to the worse survival time of glioma patients

We used the *Scissor* (v2.0) package to identify malignant cells associated with survival time (Sun et al., 2021). We conducted Scissor analysis for GSE131928, GSE70630, and GSE89567 by integrating the scRNA-seq datasets with the TCGA datasets of GBM, oligodendroglioma, and astrocytoma as follows (other scRNA-seq datasets lack the corresponding TCGA datasets). **First**, expression files and sample information of TCGA GBM and LGG were downloaded through *TCGAbiolinks* (v2.25.3). The oligodendroglioma and astrocytoma TCGA datasets are referred to as LGG because they belong to grade II low-grade gliomas. **Second**, TCGA samples without survival time and status were excluded. **Third**, we matched TCGA samples with scRNA-seq samples by age based on the following two considerations. The first one is that age is an important prognostic factor in glioma (Corell et al., 2018; Kim et al., 2021; Jia et al., 2022; Ostrom et al., 2022). For example, the age-adjusted incidence rate of GBM increases from 1.25 per 100,000 people among adults 35–34 years to 8.05, 12.99, and 15.13 among adults 55–65, 65–74, and 75–84 years, respectively (Ostrom et al., 2022); a recent finding revealed that the risk of mortality increases with age in a J-shaped pattern (Jia et al., 2022). The second consideration is that due to the limited samples in the three glioma scRNA-seq datasets, the age distribution in these datasets exhibits considerable variation and differs significantly from that of the TCGA samples. Specifically, the 20 GBM samples were between 52 and 78 years old; the 7 anaplastic astrocytoma samples were between 24 and 47 years old; the 3 oligodendroglioma samples were aged 31, 35, and 67 years old, respectively (Supplementary Table S1). In order to minimize the confounding effects of the age and tumor subtype, only TCGA samples matching corresponding scRNA-seq samples were included in the following analysis. Thus, only TCGA GBM samples aged between 52 and 78 were included for analyzing GSE131928; only TCGA oligodendroglioma samples with 1p/19q codeletion and aged between 30 and 40 or between 60 and 70 were included for analyzing GSE70630; only TCGA anaplastic astrocytoma samples with IDH1 mutation and aged between 24 and 47 were included for analyzing GSE89567. **Fourth**, for each of the three glioma types, the *Scissor* program was used to identify Scissor^+^ cells and Scissor^−^ cells, with the parameter settings “cutoff” = 0.2, “family” = “cox”, and “alpha” = 0.001/0.002/0.08 for astrocytoma/GBM/oligodendroglioma. These parameters ensured that the ratio of selected cells did not exceed 50% of the total analyzed cells. Scissor^+^ cells are associated with worse survival, while Scissor^−^ cells are associated with good survival (Sun et al., 2021). **Finally**, we used the one-sided Fisher’s exact test to investigate whether Scissor^+^ or Scissor^−^ cells were enriched with NGS^+^ cells, and *p*-value < 0.01 was defined as the significant level.

### 2.9 Detection of differentially expressed genes in NGS^+^ cells

We used the *FindMarkers* function in *Seurat* to identify differentially expressed genes (DEGs) in NGS^+^ cells against NGS^−^ cells with the parameter settings “min.pct” = 0, “logfc.thresholds” = 0.5, “test.use” = “wilcox”, and other parameters adopting the default values). Genes with logFC > 0.5 and adjusted *p*-value < 0.01 were defined as DEGs. To reveal what the *Biological Process* gene ontology (GO) terms DEGs are related to, gene set enrichment analysis was performed using the *ClusterProfiler* (v3.14.3) R package (with default parameters, BH-adjusted *p*-value < 0.05) (Yu et al., 2012).

### 2.10 Prediction of transcriptional regulation of NGS genes by lncRNAs

To analyze NGS gene regulation by lncRNAs, we jointly used two methods on the transcriptome and genome levels, respectively. The first was the computation of Bayesian correlation between each pair of lncRNAs and NGS genes using the *Baco. R* function (with default parameters) (https://github.com/dsancheztaltavull/Bayesian-Correlations/blob/master/BaCo.R). Bayesian correlation is a robust measure for correlated genes in scRNA-seq data (Sanchez-Taltavull et al., 2020). The second was the prediction of lncRNA/DNA binding using the *LongTarget* program (with default parameters, all DBSs > 50 bp) (He et al., 2015; Lin et al., 2019). Many lncRNAs can bind to specific DNA sequences and recruit DNA and histone modification enzymes to the DNA binding sites. *LongTarget* predicts DNA binding domains (DBDs) in lncRNAs and DNA binding sites (DBSs) in DNA sequences simultaneously. We predicted the DBSs of lncRNAs in the promoter regions (+3,500 bp ∼ −1,500 bp upstream and downstream transcription start site) of NGS genes. If a lncRNA and an NGS gene have a Bayesian correlation > 0.4 (adjusted *p*-value < 0.05) and the lncRNA has a DBS with binding affinity > 100 (about > 150 bp) in the promoter region of the NGA gene, the lncRNA was assumed to be able to regulate the NGS gene. The networked regulations of multiple NGS genes by multiple lncRNAs were visualized using the *Cytoscape* program (3.9.1).

### 2.11 Validation of the analysis methods and results

We validated the lncRNA/DNA binding prediction using two methods. First, we identified human-specific lncRNAs from the 13562 GENCODE-annotated human lncRNAs, predicted their DBSs genome-wide using the *LongTarget* program (He et al., 2015; Lin et al., 2019), used the CRISPR/Cas9 technique to knock out the predicted DBDs in three human-specific lncRNA genes in the HeLa cell line, RKO cell line, and SK-MES-1 cell line, and performed RNA-seq before and after the DBD knockout, then analyzed DEGs in each cell line. Second, the genome-wide DNA binding sites of NEAT1, MALAT1, and MEG3 were experimentally identified using ChIRP-seq (West et al., 2014; Mondal et al., 2015), we predicted the genome-wide DBSs of the three lncRNAs (NEAT1, MALAT1, and MEG3, compared predicted DBSs with the experimentally identified ones and examined whether the two groups overlap significantly (Wen et al., 2022).

We validated the contribution of NGS^+^ cells to the poor survival of glioma patients by performing two survival analyses. First, expression files and sample information of TCGA GBM and LGG were downloaded through *TCGAbiolinks* (v2.25.3). TCGA samples without survival information were discarded. To remove the confounding effect of sex and age, we set these factors as fixed effects in the *coxph* function. Using the *Scissor* program (Sun et al., 2021), we then identified malignant cells associated with patient survival time. We used Fisher’s exact test to examine whether the Scissor + cell group was predominantly composed of NGS + cells. Second, to verify that lncRNAs involved in regulating NGS genes influence survival time, we used the Cox proportional hazards model (in the R package *survival* v3.2.7) to examine whether lncRNAs regulating NGS genes correlate with survival time.

XIST is an important lncRNA and regulates many genes genome-wide (Statello et al., 2021). We observed that XIST has DBSs in 12 out of 15 NGS genes in the oligodendroglioma dataset. To examine whether these DBSs could be determined by chance, we conducted a permutation test. First, we predicted XIST’s DBSs genome-wide. Of all the 45,515 annotated genes examined, 13,504 genes have DBSs. Second, we randomly sampled 15 genes from the whole genome and calculated the ratio of genes having DBS of XIST, repeated the process 1,000,000 times, and obtained an empirical distribution of this ratio. Finally, we compared 12 out of the 15 NGS genes with this distribution and obtained the *p*-value.

## 3 Results

### 3.1 NGS gene expression characterizes glioma types, cell clusters, and patients

We downloaded nine scRNA-seq datasets of five gliomas from the GEO website, including glioblastoma (GBM), diffuse H3K27M glioma (H3K27M glioma), medulloblastoma (MB), 1p/19q codeleted oligodendroglioma (oligodendrocyte or OD), and IDH1-mutant anaplastic astrocytoma (astrocytoma or IDH1-mutant AA) (Supplementary Table S1). We integrated NGS genes in these datasets (Supplementary Table S2). Upon gene annotations, these NGS genes are involved in microtube assembly, synaptogenesis, neurotransmitter receptors, and neuronal paracrine based on the experimental studies (Venkataramani et al., 2019; Venkatesh et al., 2019; Zeng et al., 2019).

**FIGURE 1:**
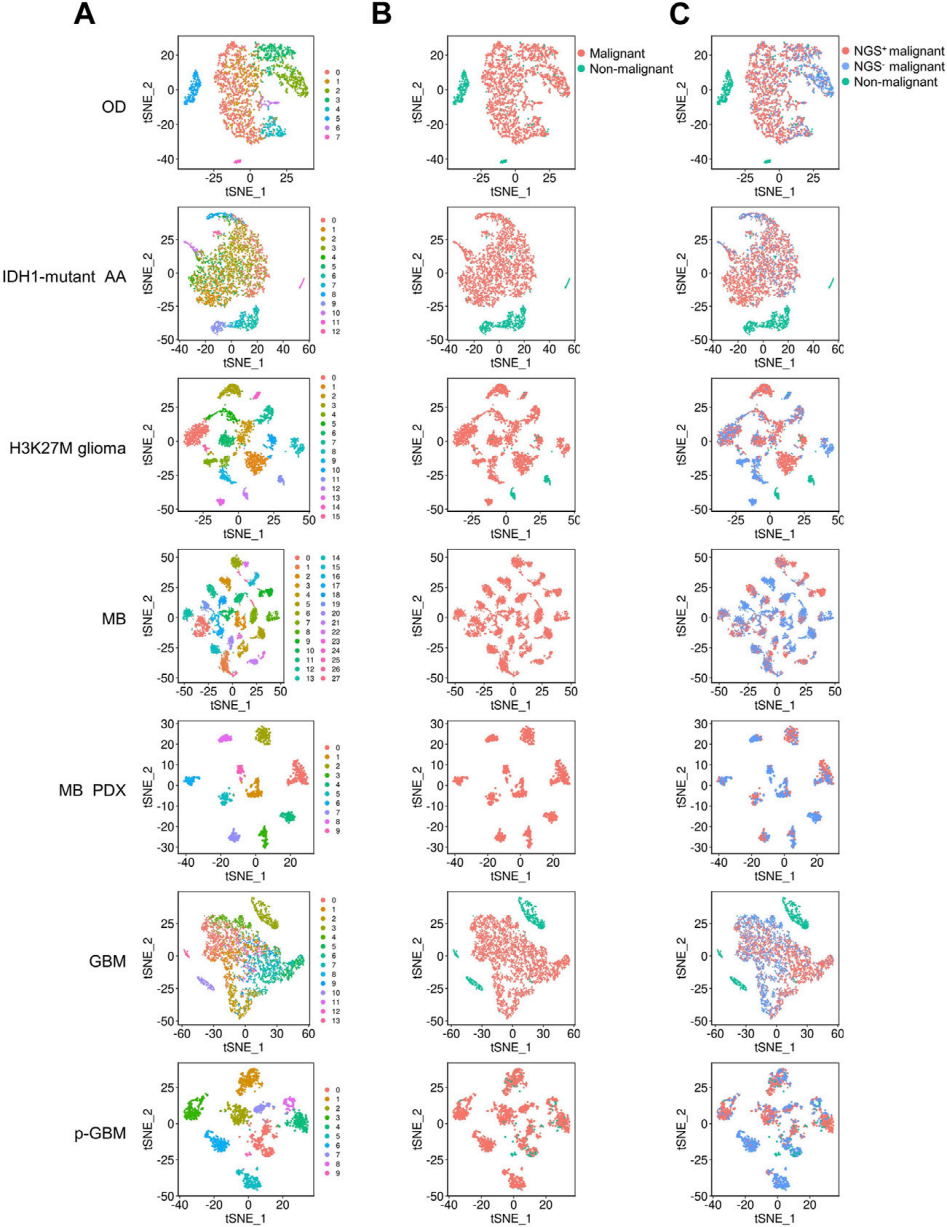
tSNE plots show the clustering of cells in the 9 glioma datasets. OD, IDH1-mutant AA, H3K27M glioma, MB, MB PDX, GBM, and p-GBM indicates 1p/19q codeleted oligodendroglioma, IDH1-mutant anaplastic astrocytoma, diffuse H3K27M glioma, medulloblastoma, patient-derived xenografts model of medulloblastoma, glioblastoma, and pediatric GBM, respectively. Each glioma consists of multiple cell clusters. **(A)** The distribution of cell clusters (numbers indicate different clusters). **(B)** The distribution of malignant and non-malignant cells. **(C)** The distribution of NGS^+^ malignant cells, NGS^−^ malignant cells, and non-malignant cells.

For each dataset, we first identified normal cells using two methods. The first method includes clustering cells into clusters, identifying DEGs in each cluster, and checking whether DEGs in a cluster are enriched in annotated marker genes of cell types collected in the *CellMarker* database (Zhang et al., 2019). If DEGs in a cell cluster were significantly enriched in the annotated marker genes of a cell type, this cell cluster was defined as the cell type and identified as a normal cell type (all cell types in the *CellMarker* database are normal cells) (Figures 1A, B). In the medulloblastoma and medulloblastoma PDX mouse models, no cell cluster was identified as normal cells. The second method includes extracting the properties of each identified normal cell cluster to refine normal cell identification. We used a method to detect “binary markers” for each normal cell cluster (Hodge et al., 2019). In this method, a binary score ranging from 0 to 1 was computed to assess the exclusivity of a DEG between clusters. A DEG in a cell cluster with a binary score > 0.7 was defined as a binary marker of that cell cluster. Upon each set of binary markers, all cells in a dataset (but not in any identified cluster) were scored. If a cell was scored above a dataset-specific cutoff, the cell was classified into the corresponding normal cell cluster.

Then, we determined whether a cell was a malignant cell jointly upon three criteria: a) It did not belong to any normal cell cluster, b) it could not be defined as a normal cell when scored by binary markers of normal cell clusters, and c) copy number analysis (CNA) revealed that it underwent abnormal genome duplication. Since no normal cells were identified in the medulloblastoma and medulloblastoma PDX mouse models, all cell clusters were malignant cells. In these gliomas, malignant cells can be divided into multiple clusters (Figures 1A, B), and some malignant cell clusters are present only in very few patients (Supplementary Table S3). For example, malignant cells in GBM were divided into 14 clusters, with clusters 7, 8, 9, and 12 comprising cells from a single patient. Thus, gliomas have high inter- and intra-tumor heterogeneity, as previously reported (Patel et al., 2014; Neftel et al., 2019).

**FIGURE 2:**
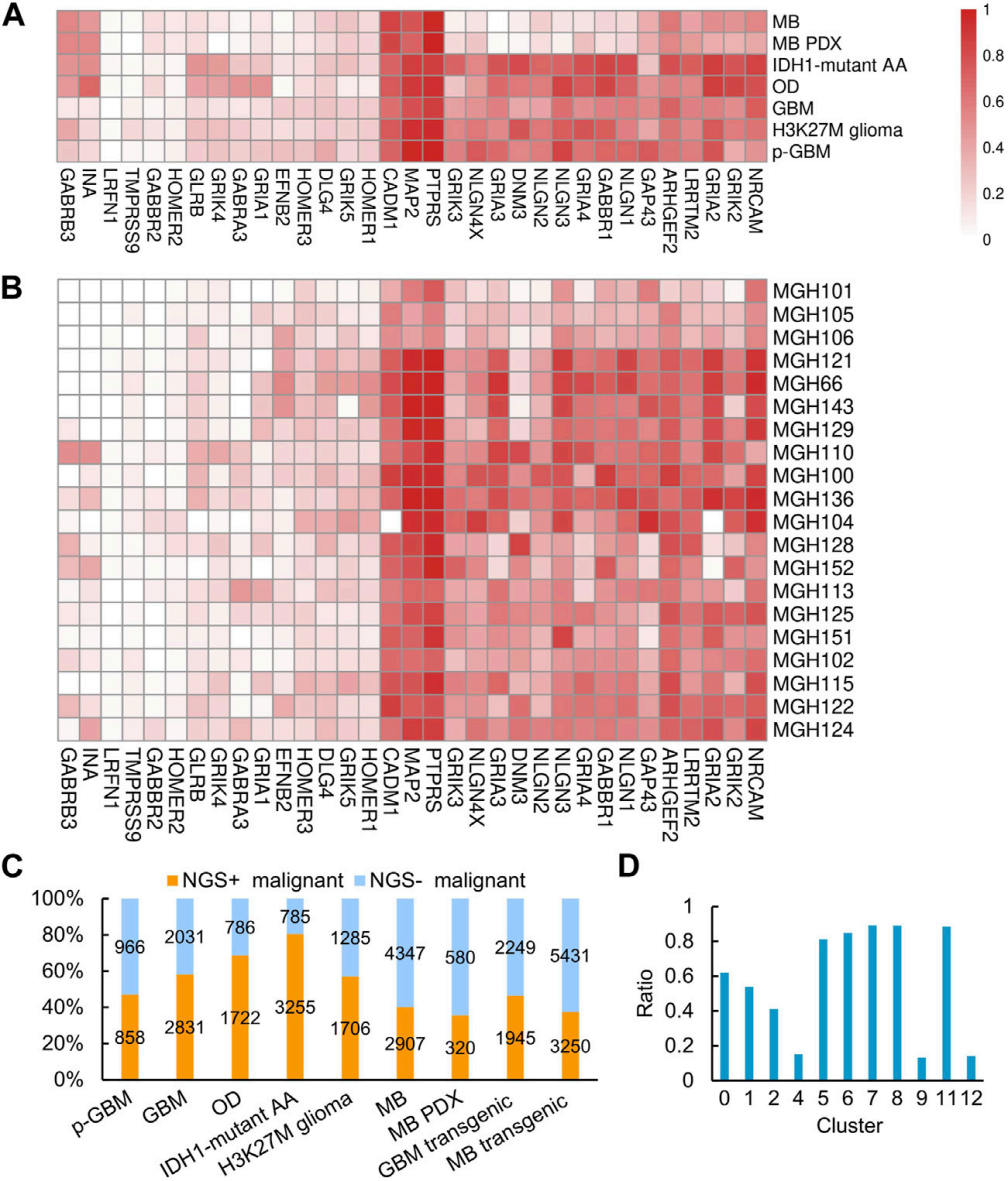
NGS gene expression in different gliomas, GBM samples, and cell clusters. **(A)** NGS gene expression in different gliomas. **(B)** NGS gene expression in different GBM samples. In **(A, B)**, the color bar indicates the percentage of expressed cells. **(C)** The percentages and numbers of NGS^+^ and NGS^−^ malignant cells in different gliomas. **(D)** The percentages of NGS^+^ cells in the 13 clusters of malignant GBM cells.

Since gliomas are highly heterogenous, we next examined whether NGS gene expression is highly heterogeneous. We found that, generally, NGS genes were expressed tumor-specifically, with distinct expression in different tumors and even in patients with the same tumor. Nevertheless^,^ the NGS genes *PTPRS*, *MAP2*, and *CADM1* were expressed in most malignant cells across these gliomas (Figures 2A, B), consistent with the observed neurological associations of these genes (Zhou et al., 2015; Hendriks et al., 2018; Pan et al., 2020). To examine whether NGS genes characterize malignant cells, we scored malignant cells upon NGS gene expression and divided malignant cells into NGS-negative (NGS^−^) and NGS-positive (NGS^+^) ones (Figure 1C). In each glioma, the NGS score in malignant cells is significantly higher than the NGS score in non-malignant cells (*p*-value = 3.664e-06), suggesting reasonable identification of malignant and normal cells. The ratio of NGS^+^ malignant cells varies not only among different types of gliomas (Figure 2C) but also among different cell clusters (Figure 2D). For example, the ratio of NGS^+^ cells in GBM in clusters 4, 9, and 12 is much lower than the ratio in other clusters (Figure 2D). Thus, different subsets of NGS genes characterize glioma types, cell clusters, and aggression ability.

### 3.2 Dysregulated genes in malignant NGS^+^ cells are enriched in neurodevelopment- and synapse-related GO terms

**FIGURE 3:**
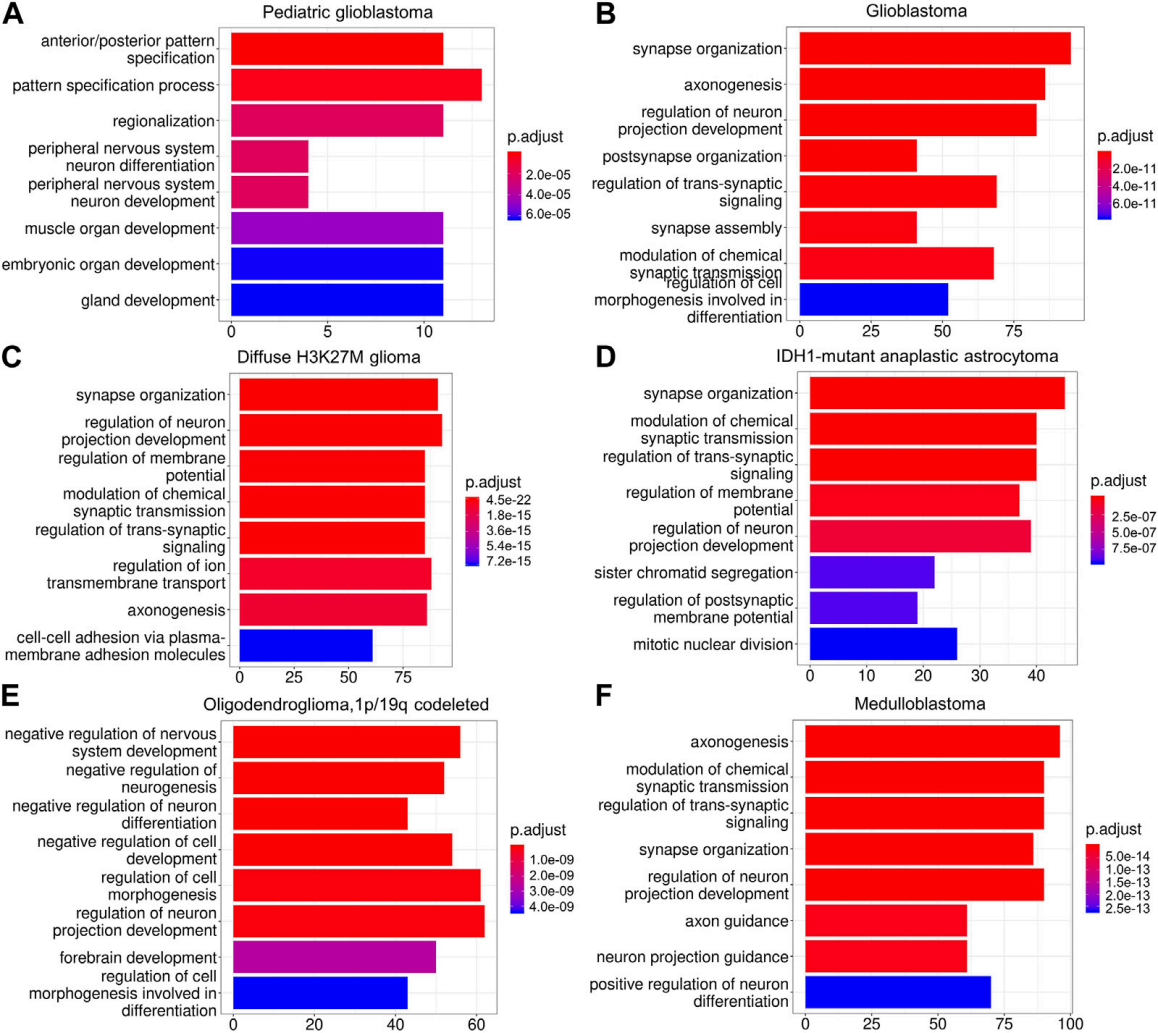
The enriched gene sets of DEGs in different gliomas **(A–F)**. DEGs are enriched in neurodevelopmental-related GO terms in all gliomas **(A–F)** and synapse-related GO terms in four gliomas **(B,C,D,F)**. These results support the importance of dysregulated NGS genes for gliomas.

Based on our definition, the critical difference between NGS^+^ malignant and NGS^−^ malignant cells is whether a malignant cell can form synapses with neurons. Thus, if our method of classifying malignant cells into NGS^+^ and NGS^−^ groups is valid, DEGs between NGS^+^ and NGS^−^ malignant cells should have an association with synapse formation. To verify this, we first identified DEGs between NGS^+^ and NGS^−^ malignant cells in each glioma (Supplementary Table S4), then applied gene set enrichment analysis to DEGs using the *ClusterProfiler* program and the Gene Ontology (GO) database. DEGs in all gliomas are significantly enriched in neurodevelopmental-related GO terms, and DEGs in many gliomas are enriched in synapse-related GO terms (Figure 3), supporting a strong association between neurons and NGS^+^ malignant cells. In addition, different gliomas show somewhat different GO terms and levels of enrichment, suggesting differences in NGS formation in different gliomas. These results also support the validity of classifying malignant cells into NGS^+^ and NGS^−^ cells.

### 3.3 LncRNAs transcriptionally regulate NGS gene expression

As abundant lncRNA genes are expressed in the human brain, it is sensible to conjecture that they also contribute to tumorigenesis in the brain. We identified differentially expressed lncRNA genes between NGS+ and NGS- malignant cells to reveal the contribution. Multiple top differentially expressed lncRNA genes (Supplementary Table S4), including LINC00599, MEG3, and TTTY15, were identified in NGS^+^ malignant cells. We next examined the tumor-specificity of lncRNA expression because lncRNA gene expression is highly tissue-specific. Distinctively dysregulated lncRNAs in gliomas include LOC150622 and LINC00645 in glioblastoma, LOC100216479 in diffuse H3K27M glioma, FLJ41350 and FTX in medulloblastoma, TPTEP1 and LOC100144595 in astrocytoma, and LOC100216545, FAM66B, and LOC100499405 in oligodendroglioma.

**FIGURE 4:**
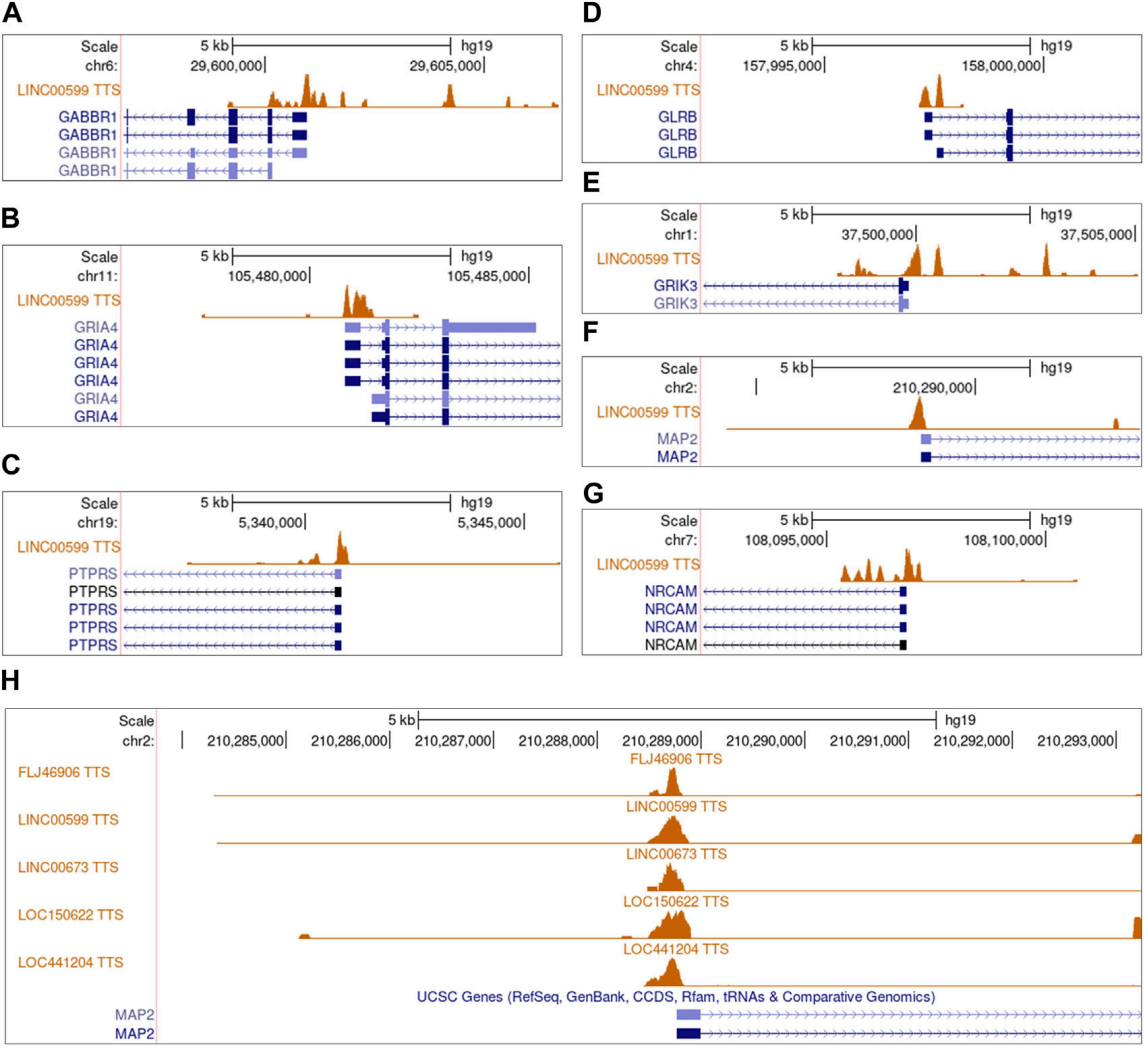
The predicted DNA binding sites (DBSs) of lncRNAs in the promoter regions of differentially expressed NGS genes. Panels are snapshots of the UCSC Genome Browser wherein predicted DBSs were uploaded as a custom track. “TTS” means triplex-targeting sites, and a set of overlapping TTSs forming a peak was a DBS. **(A–G)** The DBSs of LINC00599 in the promoter region of multiple differentially expressed NGS genes. The peaks in each panel’s “LINC00599 TTS” track indicate LINC00599s DBSs. **(H)** The DBSs of FLJ46906, LINC00599, LIC00673, LOC150622, and LOC441204 in the promoter region of *MAP2*.

To obtain evidence supporting that dysregulated lncRNAs regulate NGS gene expression, we used two methods to examine the relationships between lncRNAs and NGS genes. On the transcriptome level, we calculated the pairwise Bayes correlation between all differentially expressed NGS genes and all differentially expressed lncRNAs. On the genome level, we used the *LongTarget* program to predict differentially expressed lncRNAs’ DNA binding sites (DBS) in the promoter regions of differentially expressed NGS genes (He et al., 2015; Lin et al., 2019). Jointly upon the two methods, 28 lncRNAs were identified to regulate NGS genes in gliomas (Supplementary Table S5). The most notable lncRNA is LINC00599, which has DBSs in many NGS genes in most gliomas, including *GABBR1*, *NRCAM, PTPRS, GLRB, GRIA4, GRIK3*, and *MAP2* (Figures 4A–G), and the most notable NGS gene is *MAP2*, which is associated with microtube assembly and regulated by multiple lncRNAs through the same DBS (Figure 4H) (Osswald et al., 2015).

### 3.4 NGS gene regulation by lncRNAs is species- and tumor-specific

**FIGURE 5:**
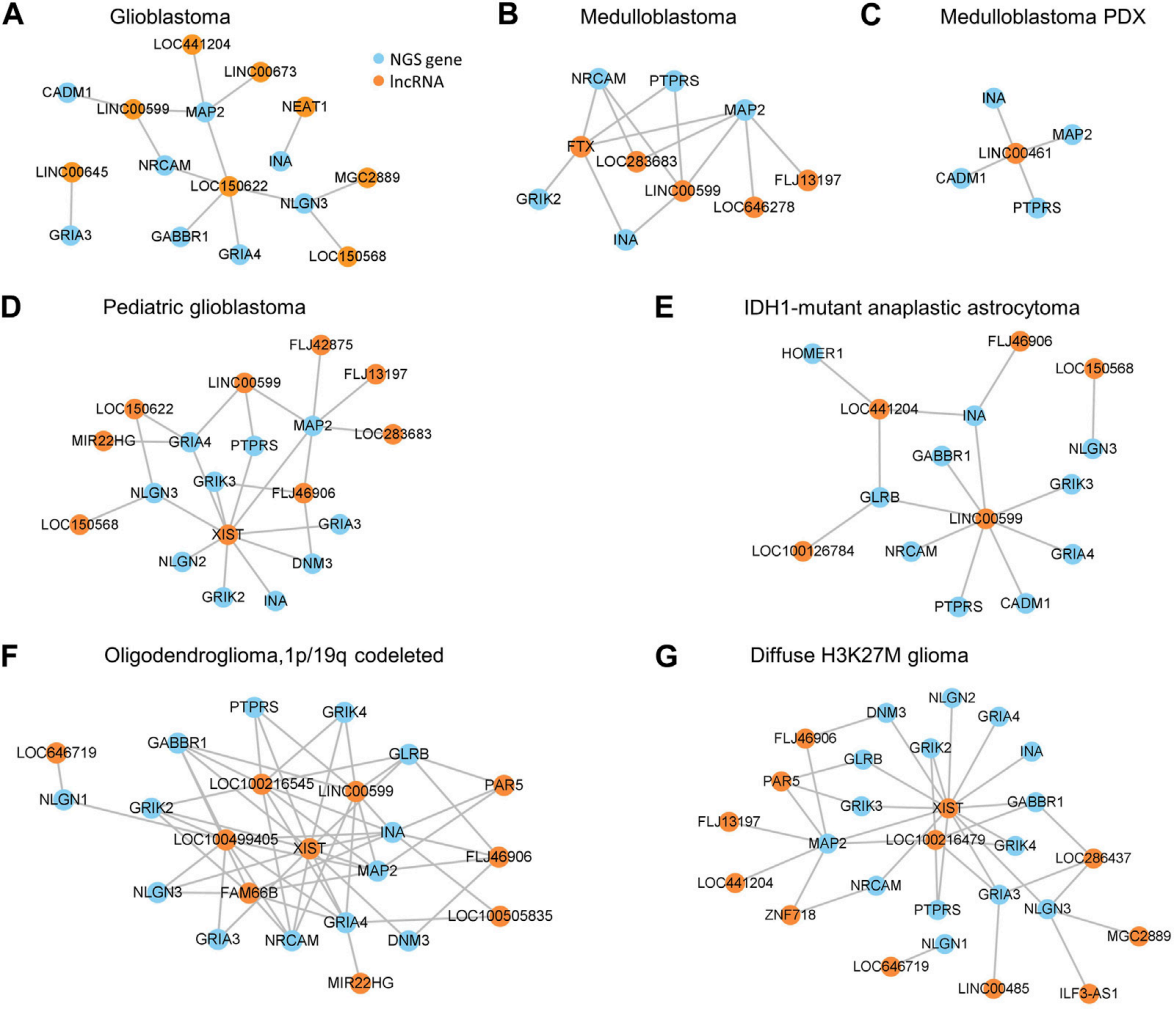
Networks of NGS gene regulation by lncRNAs based jointly on Bayes correlation and lncRNA-target gene relationship. **(A–G)** The networks in GBM **(A)**, MB **(B)**, MB PDX **(C)**, p-GBM **(D)**, IDH1-mutant AA **(E)**, OD **(F)**, and H3K27M glioma **(G)**.

Many studies reported tissue-specific expression of lncRNA genes in tumors (Mattioli et al., 2019; Liu et al., 2021)^,^ suggesting their tumor-specific regulatory functions. To examine whether tumor-specific lncRNA gene expression causes tumor-specific NGS gene expression, we generated the regulatory networks of lncRNAs and NGS genes in different gliomas based jointly on the Bayes correlation and predicted lncRNA-target NGS gene relationship (Figure 5). **First**, 61% of lncRNAs regulate NGS genes in one specific glioma (Supplementary Table S5), suggesting that most lncRNAs regulate NGS genes tumor-specifically. **Second**, some essential NGS genes such as *MAP2* are regulated by different lncRNAs in different gliomas; meanwhile, important lncRNAs such as LINC00599 regulate different NGS genes in different gliomas (Figure 5). **Third**, an NGS gene can be regulated by different lncRNAs in humans and mice. Notable examples are *MAP2*, *PTPRS*, and *INA* which are expressed in MB and mouse MB PDX; these genes are regulated by multiple lncRNAs in human MB but only by LINC00461 in mouse MB PDX. Similarly, a lncRNA can target different NGS genes in humans and mice. We found that LINC00461 regulates NGS genes in mouse MB PDX but does not regulate any NGS gene in human gliomas. These suggest that NGS gene regulation by lncRNAs relies on specific transcriptional landscapes, and such landscapes may critically characterize patterns of NGS gene regulation. In support of this interpretation, Miat was found to regulate the NGS gene *Gria2* in transgenic mouse MB, but MIAT (the human orthologue of Miat) was found not to regulate any NGS genes in these glioma datasets (Supplementary Table S5). **Fourth**, NGS genes in mouse MB PDX showed similar expression levels to NGS genes in human MB; however, NGS genes in mouse transgenic MB showed different expression levels from NGS genes in human GBM (Supplementary Table S6). **Fifth**, the identified networks indicate that LINC00599 is a critical lncRNA regulating NGS genes in multiple gliomas, with LINC00599-MAP2 and LINC00599-NRCAM being the core lncRNA-NGS gene pairs. These results suggest that NGS gene regulation by lncRNAs is highly species- and tumor-specific, and these lncRNAs provide a new class of targets for glioma treatment.

### 3.5 Simian-specific lncRNAs are more involved in NGS gene regulation

**FIGURE 6:**
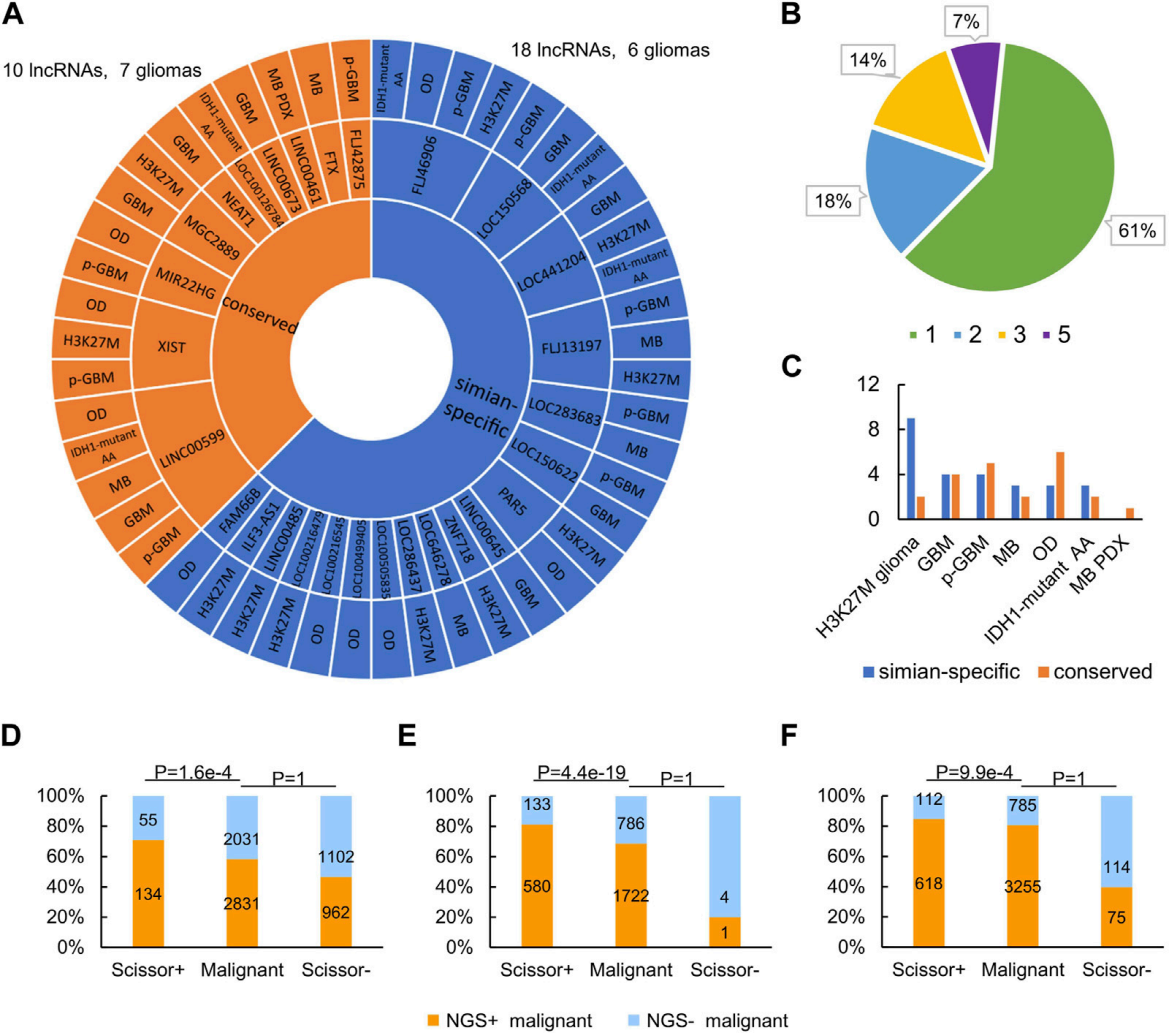
NGS regulation by lncRNAs has significant cross-species differences. **(A)** Conserved and simian-specific lncRNAs that regulate NGS genes in gliomas. **(B)** The percentages of lncRNAs that regulate NGS genes in 1, 2, 3, and 5 gliomas. **(C)** Numbers of conserved and simian-specific lncRNAs in each glioma. **(D–F)** Compared with the malignant cell group (as the background), the Scissor^+^ cell group is significantly enriched in NGS^+^ cells, while the Scissor^−^ cell group is significantly lacking in NGS^+^ cells, in GBM **(D)**, oligodendroglioma **(E)**, and IDH1-mutant anaplastic astrocytoma **(F)** (*p*-value = 1.6e-4, 4.4e-19, and 9.9e-4, respectively, Fisher’s exact test).

Clade-specific lncRNAs, such as simian- and rodent-specific ones, are evolutionarily younger than lnRNAs conserved in mammals. Since simian-specific lncRNAs in the human genome have contributed specifically to the evolution of the human brain (Derrien et al., 2012; Briggs et al., 2015; Rashighi and Harris, 2017; Zimmer-Bensch, 2019), it is sensible to conjecture that these lncRNAs may also greatly affect brain tumorigenesis compared with lncRNAs conserved in mammals. To test this conjecture, we examined the conservation of the 28 lncRNAs that regulate NGS genes in these gliomas (Supplementary Table S5). Upon the whole-genome alignments in the UCSC Genome Browser (https://genome.ucsc.edu) and the simian-specific lncRNAs in the lncRNA database *LongMan* (http://www.gaemons.net/LongMan) (Lin et al., 2019), we found that 10 (35.7%) of the 28 lncRNAs are conserved in mammals (i.e., having orthologues in non-primate mammals) but 18 (64.3%) of the 28 lncRNAs are simian-specific (i.e., having orthologues only in marmoset, macaque, and chimpanzee) (Figure 6A). Additionally, compared with the ratio of all simian-specific lncRNAs to all ENCODE-annotated human lncRNAs, the ratio of NGS gene regulation-related simian-specific lncRNAs to all NGS gene regulation-related lncRNAs is significantly higher (*p*-value = 0.047, Fisher’s exact test). This suggests that simian-specific lncRNAs not only affect tumorigenesis in the brain but also determine the differences of gliomas in humans and mice. This lends an explanation for the finding that NGS gene regulation by lncRNAs is highly species-specific. In accordance, in the 17 (61%) lncRNAs that regulate NGS genes in one glioma (Figure 6B), 11 are simian-specific.

Many human diseases have distinct evolutionary roots in the genome (Maxwell et al., 2014; Benton et al., 2021), and the same may be true for brain tumors. By checking lncRNAs regulating NGS genes in different glioma, we found that most lncRNAs in diffuse H3K27M glioma are simian-specific and most lncRNAs in OD are conserved in mammals (Figure 6C). This probably highlights different evolutionary origins of epigenetic regulation of genes in different types of gliomas.

### 3.6 Validation of analysis methods and results

A key component of the above human/mouse comparative pan-gliomas analyses is analyzing the regulation of NGS genes by lncRNAs through predicting DBDs in lncRNAs and their DBSs in NGSs genes. Thus, we validated DBD/DBS prediction in three ways. **First**, in a related study, we identified human-specific lncRNAs from the 13562 GENCODE-annotated human lncRNAs (http://www.gaemons.net/LongMan) and predicted the genome-wide DBSs of these lncRNAs (He et al., 2015; Lin et al., 2019). We used the CRISPR/Cas9 technique to knock out the predicted DBDs in three human-specific lncRNA genes in the HeLa, RKO, and SK-MES-1 cell lines and performed RNA-seq before and after the DBD knockout. Differential gene expression analysis revealed that the |fold change| of target genes (DEGs whose promoter regions contain DBSs of these lncRNAs) was significantly larger than the |fold change| of non-target genes (DEGs whose promoter regions do not contain DBSs of these lncRNAs) (one-sided Mann-Whitney test, *p*-value = 3.1e-72, 1.49e-114, and 1.12e-206 for RP13-516M14.1, RP11-426L16.8, and SNORA59B) (see *Data availability statement*). These results suggest that the knockout of DBDs caused the changed expression of target genes. **Second**, since the genome-wide DNA binding sites of NEAT1, MALAT1, and MEG3 were experimentally identified using ChIRP-seq (West et al., 2014; Mondal et al., 2015), we predicted genome-wide DBSs of NEAT1, MALAT1, and MEG3, compared predicted DBSs with the experimentally identified one, and found that the two groups of DBSs overlap well (Wen et al., 2022). **Third**, the *LongTarget* program uses a variant of the Smith-Waterman algorithm to identify all local alignments between a pair of RNA and DNA sequences, thus identifying DBDs and DBSs simultaneously. We computed the likelihood that an alignment could be generated by chance. According to the alignment algorithm, a DBS of >140 bp is extremely unlikely to be generated by chance (*p* < 8.2e-19 to 1.5e-48). These results support the analysis of NGS gene regulation by lncRNAs.

Malignant cells in gliomas hijack potassium currents to promote their aggression and proliferation, suggesting that glioma patients with substantial NGS formation should have a worse prognosis. Therefore, we performed two survival analyses of glioma patients. **First**, we downloaded the TCGA data of GBM, oligodendroglioma, and astrocytoma (the three gliomas have TCGA datasets) and identified malignant cells associated with patient survival time using the *Scissor* program (Sun et al., 2021). *Scissor* was developed to associate cells in scRNA-seq data with some trait (here, the survival time) in the corresponding TCGA data. According to the usage of *Scissor*, Scissor^+^ cells were defined as cells associated with worse survival, while Scissor^−^ cells were defined as cells associated with good survival. We used Fisher’s exact test to examine whether the Scissor^+^ cell group was predominantly composed of NGS^+^ cells. Compared with the malignant cell group as the background, the Scissor^+^ cell group is significantly enriched in NGS^+^ cells, while the Scissor^−^ cell group is significantly lacking in NGS^+^ cells in GBM, oligodendroglioma, and astrocytoma (Figures 6D–F; *p*-value = 1.6e-4, 4.4e-19, and 9.9e-4, respectively). Specifically, the *Scissor* program identified 189 Scissor^+^ cells (including 55 NGS^−^ cells and 134 NGS^+^ cells) and 2064 Scissor^−^ cells (including 1102 NGS^−^ cells and 962 NGS^+^ cells) in GBM, 713 Scissor^+^ cells and 5 Scissor^−^ cells in oligodendroglioma, and 730 Scissor^+^ cells and 189 Scissor^−^ cells in astrocytoma (Figures 6D–F). In line with the relationship between NGS formation and survival time, these results support that NGS^+^ cells contribute to the worse survival of glioma patients. **Second**, to verify that lncRNAs involved in regulating NGS genes influence survival time, we used the Cox proportional hazards model (in the R package *survival* v3.2.7) and lncRNAs to perform survival analysis. The analysis unveiled that 5 of 28 lncRNA potentially regulating NGS genes were correlated with survival. Among them, the lower LINC00599 expression and higher XIST expression were significantly correlated with worse survival of low-grade glioma patients (Supplementary Table S7). This result is supported by a related study (Fu et al., 2019).

Finally, we conducted a permutation test using the lncRNA XIST and its target NGS genes in the oligodendroglioma dataset to check whether NGS gene regulation by lncRNAs could occur by chance. XIST has DBSs in 12 out of 15 NGS genes in the oligodendroglioma dataset and has DBSs in 13,504 genes out of the 45,515 Ensembl-annotated genes. We randomly sampled 15 genes from the whole genome, calculated the ratio of genes having DBS of XIST, repeated the process 1,000,000 times, obtained an empirical distribution of this ratio, and compared 12 out of the 15 NGS genes with the obtained distribution. The result suggests that NGS gene regulation by XIST unlikely occurs by chance (*p*-value = 8e-5, permutation test).

## 4 Discussion

Gliomas are the most lethal tumors in humans. NGS in gliomas and brain-metastatic breast cancers were recently found to promote tumor progression (Venkataramani et al., 2019; Venkataramani et al., 2022b; Venkatesh et al., 2019; Zeng et al., 2019). This finding greatly improves the understanding of why gliomas are highly aggressive and highlights the potential of targeting NGS as a treatment for gliomas. While NGS genes have drawn increasing attention (Ji et al., 2020; Lin et al., 2021), some questions remain unclear, including whether NGS gene expression shows species- and tumor-specificity and whether lncRNAs regulate the specificity in a species-specific manner. Since targeting NGS genes (which have wide functions) may cause undesired side effects, and researchers use PDX and transgenic mouse models to study gliomas, answering the two questions and exploring targetable lncRNAs are important.

This study addresses these questions by checking NGS gene expression and regulation by lncRNAs in multiple gliomas in humans and mice. The main results are that lncRNAs regulate NGS genes highly species- and tumor-specifically and that simian-specific lncRNAs in humans are more involved in NGS gene regulation than conserved lncRNAs. These findings, with Supplementary Material, suggest that lncRNAs are potential tumor-specific targets for human gliomas. To verify that lncRNAs involved in regulating NGS genes influence the survival time of glioma patients, we combined scRNA-seq data and TCGA data to perform survival analyses using two methods. These analyses generated consistent results.

Two studies examined 17 and 231 synapse-related genes in human gliomas (Ji et al., 2020; Lin et al., 2021). Compared with them, our cross-species pan-gliomas analysis focused on 34 NGS genes collected from the literature. Despite using different genes, all of these studies demonstrate the prognostic values of NGS genes. Previous studies have also revealed that thousands of new lncRNAs have emerged during primate nervous system evolution and that nearly half of the identified lncRNAs are specifically expressed in the human brain (Derrien et al., 2012; Rashighi and Harris, 2017; Zimmer-Bensch, 2019). Of interest, we found that simian-specific lncRNAs are more involved in NGS gene regulation than lncRNAs conserved in mammals. Thus, simian-specific lncRNAs contribute not only to the evolution of the brain but also to the development of brain tumors. We also observed that different types of gliomas may have different evolutionary origins. For instance, most lncRNAs regulating NGS genes in diffuse H3K27M glioma are simian-specific, while most lncRNAs regulating NGS genes in OD are conserved in mammals. This finding supports the recent viewpoint that many human diseases have distinct evolutionary roots in the genome (Maxwell et al., 2014; Benton et al., 2021).

Some comparative cancer studies suggested conservation between human and mouse tumors (Aytes et al., 2014; Onken et al., 2014; Woo et al., 2021). However, many drugs tested successfully in mice failed in human clinical trials (Gould et al., 2015). We observed that, although NGS genes show a similar expression pattern in PDX mouse models, which is consistent with the finding that more than 80% of glioma cells in PDX mouse models had tumor microtubes (Drumm et al., 2020), the patterns of NGS gene expression in transgenic mouse glioma models and human gliomas are poorly comparable. When it comes to the regulation of NGS gene by lncRNAs, neither PDX mouse models nor transgenic mouse models share commonalities with human gliomas. This agrees with the finding that phenotypic differences between human and mouse orthologous genes are mainly caused by the evolutionary rewiring of regulatory networks (Ha et al., 2022). In brief, this study reveals that lncRNAs regulate NGS genes highly species- and tumor-specifically and that simian-specific lncRNAs are substantially involved in human gliomas. These findings help rational design of mouse models for glioma studies (Landgraf et al., 2018), and suggest that caution is needed when interpreting discoveries from glioma studies using PDX and transgenic mouse models.

## 5 Conclusion

NGS genes are regulated by lncRNAs and this regulation is highly species- and tumor-specific. NGS gene expression and regulation in human gliomas are quite different from those in PDX and transgenic mouse models. Simian-specific lncRNAs with tumor-specific expression and function provide a new class of potential targets for glioma treatment.

## Data Availability

The original contributions presented in the study are included in the article/Supplementary Material. Human lncRNAs’ orthologues in 16 mammals and the *LongTarget* program are available at the *LongTarget/LongMan* website (http://www.gaemons.net/). The RNA-seq data of the three HS lncRNAs before and after the CRISPR/Cas9 experiments have been deposited in the NCBI GEO database (https://www.ncbi.nlm.nih.gov/geo) under the accession number GSE213231. Further inquiries can be directed to the corresponding authors.

## Abbreviations

AA, anaplastic astrocytoma; DBD, DNA binding domains; DBS, DNA binding site; DEG, differentially expressed gene; GBM, glioblastoma; GO, Gene Ontology; MB, medulloblastoma; NGS, neuron-glioma synapse; NGS gene, neuron-glioma synapse related gene; OD, 1p/19q codeleted oligodendroglioma; PDX, patient-derived xenografts model; p-GBM, pediatric GBM; TTS, triplex-targeting site.
